# Idiopathic cutaneous Mastocytosis mimicking infantile psoriasis in a six-month-old male: a rare presentation

**DOI:** 10.1093/omcr/omaf077

**Published:** 2025-06-27

**Authors:** Carlos Diaz Q, Dana Lippmann, Diego Rios, Ricardo Andres Caravantes, Carla Perez, Ixlem Roquel

**Affiliations:** Universidad Francisco Marroquin; Universidad Rafael Landivar; Universidad Francisco Marroquin; Universidad Francisco Marroquin; Universidad Rafael Landivar; Universidad Rafael Landivar

**Keywords:** rheumatology, allergy, dermatology

## Abstract

Cutaneous mastocytosis is an uncommon condition, representing a subset of mast cell disorders primarily affecting the skin. It can manifest in various forms, including urticaria pigmentosa, mastocytomas, or diffuse cutaneous involvement. While generally benign in infants, it can mimic other dermatological conditions, such as eczema or psoriasis, leading to misdiagnosis. In resource-limited environments, diagnostic delays are further compounded by restricted access to dermatological specialists and laboratory investigations. This case underscores the clinical and socioeconomic complexities of diagnosing and managing idiopathic cutaneous mastocytosis (ICM) in a rural Guatemalan infant.

## Introduction

Idiopathic cutaneous mastocytosis (ICM) is a rare disorder in infants, characterized by the excessive proliferation of mast cells within the skin, with an annual incidence rate of 1.1 per 100 000 person-years [1]. This case describes a 6-month-old male presenting with lesions resembling infantile psoriasis on the scalp, extremities, and torso. Misdiagnosis was complicated by limited healthcare access due to the family’s disadvantaged socioeconomic background. Through clinical evaluation and histopathological confirmation, ICM was identified. The patient improved with symptomatic management, highlighting the importance of recognizing atypical dermatological manifestations of mastocytosis and addressing the challenges of healthcare inequity in resource-limited settings.

## Case presentation

**Figure 1:**
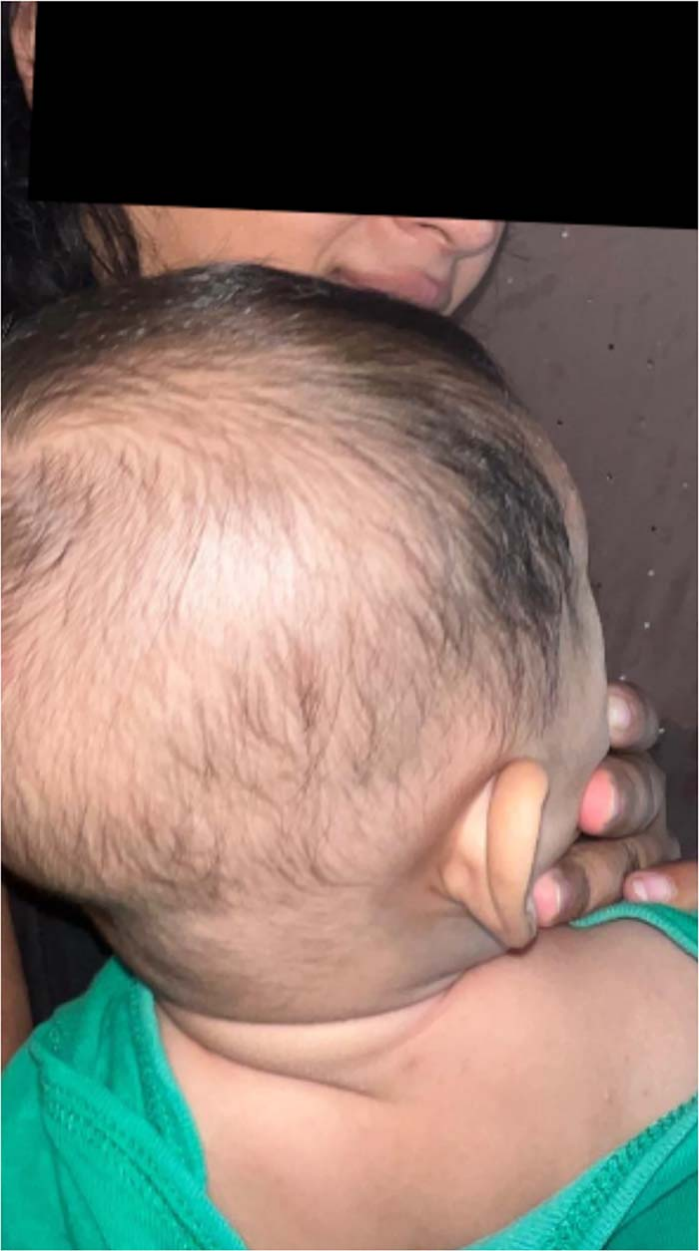
Erythematous, scaly lesions localized on the scalp, presenting as well-demarcated plaques with fine scaling. The lesions exhibit hyperpigmented borders suggestive of partial resolution. These findings were initially misdiagnosed as infantile psoriasis, highlighting the diagnostic challenge in distinguishing between idiopathic cutaneous mastocytosis and other dermatological conditions.

**Figure 2:**
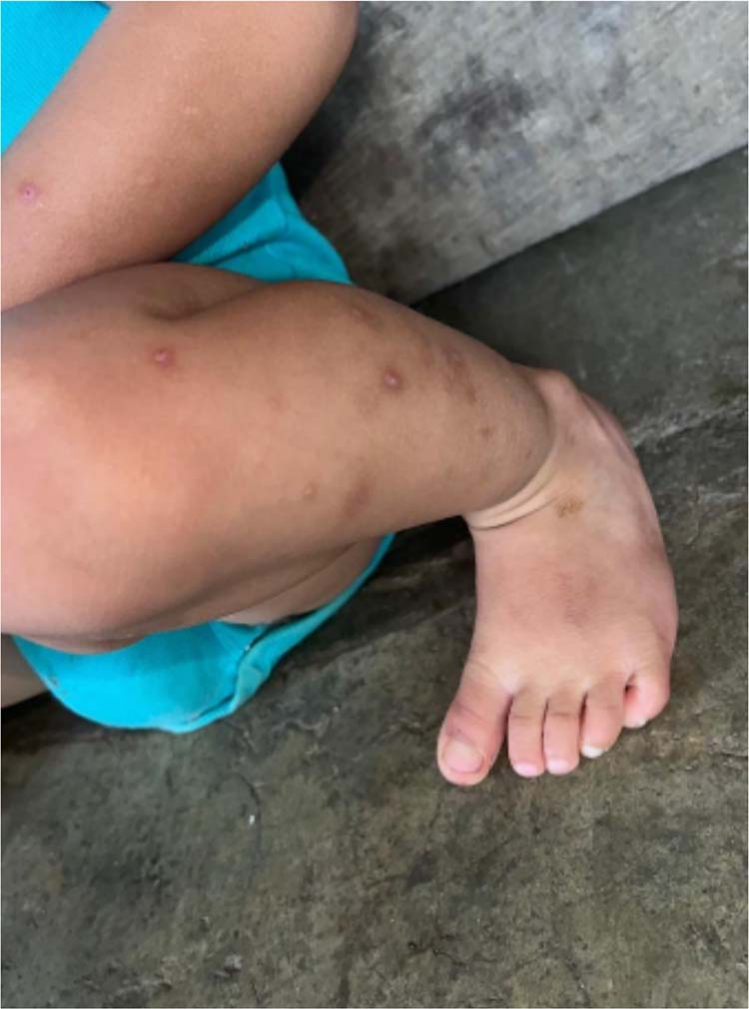
Discrete erythematous plaques on the dorsum of the foot, with a mixed pattern of maculopapular lesions and subtle scaling. The lesions demonstrated a positive Darier’s sign, with erythema and swelling upon gentle rubbing, consistent with the diagnosis of idiopathic cutaneous mastocytosis.

A 6-month-old male infant from a rural Guatemalan village presented to a local clinic with progressive skin lesions over the scalp, hands, and feet. The child was born at term via unassisted vaginal delivery, with no prenatal care due to the family’s financial constraints. The parents reported a 3-month history of recurrent, erythematous, scaly plaques initially appearing on the head and later spreading to the extremities and trunk (Figs 1 and 2). The lesions occasionally appeared pruritic, with exacerbations noted after bathing or prolonged sun exposure.

Physical examination revealed well-demarcated, erythematous plaques with overlying fine scales on the scalp, bilateral forearms, and dorsum of the feet. Similar lesions were scattered over the torso, with areas of hyperpigmentation following resolution of previous plaques. The infant appeared well-nourished with no systemic symptoms such as fever, gastrointestinal disturbances, or respiratory involvement.

The family’s rural location and economic challenges limited access to diagnostic resources. A local physician initially diagnosed the condition as infantile psoriasis and prescribed topical emollients and corticosteroids, which provided minimal improvement. The infant was subsequently referred to a community hospital, where further evaluation was performed.

Laboratory workup revealed mild eosinophilia (absolute eosinophil count: 700 cells/μl) and elevated serum tryptase levels (20 ng/ml, normal range < 11.4 ng/ml). Skin biopsy from an affected lesion on the forearm showed dense perivascular and interstitial infiltrates of mast cells, confirmed by Giemsa staining. These findings were consistent with cutaneous mastocytosis. No systemic involvement was detected. Based on clinical and histological findings, a diagnosis of idiopathic cutaneous mastocytosis was made.

## Discussion

### Differential diagnosis

ICM often mimics other dermatological conditions, particularly infantile psoriasis. In this case, the erythematous, scaly plaques were initially mistaken for psoriatic lesions, a common diagnostic pitfall. Differentiating features included the absence of nail changes, Auspitz sign, and systemic psoriatic findings. Also the presence of hyperpigmentation following lesion resolution—a hallmark of mastocytosis [2, 3].

### Pathophysiology and presentation

Cutaneous mastocytosis arises from abnormal accumulation of mast cells in the skin, driven by mutations such as KIT D816V in some cases. In infants, the condition often manifests as urticaria pigmentosa or mastocytomas, but atypical presentations resembling psoriasis, as in this case, are rare. Lesions typically exacerbate with triggers such as friction, temperature changes, or irritants, due to mast cell degranulation and release of histamine and other mediators [4].

### Management challenges in resource-limited settings

The patient’s rural residence and financial constraints delayed definitive diagnosis and management. Limited access to dermatopathology services needed referral to a regional center for biopsy and specialized staining. Additionally, the cost of advanced diagnostic tools, such as serum tryptase testing, posed significant barriers [5].

Management in this setting focused on symptomatic relief, including oral antihistamines (diphenhydramine) for pruritus and avoidance of known triggers. The family was counseled extensively on proper skin care, use of emollients, and environmental precautions. Despite financial challenges, the infant demonstrated marked improvement within four weeks of consistent management [6].

### Prognosis

Cutaneous mastocytosis in infants is generally self-limiting, with spontaneous regression often occurring by adolescence. However, long-term follow-up is essential to monitor for potential progression to systemic mastocytosis or persistence of cutaneous symptoms [7].

## Conclusion

This case illustrates the diagnostic challenges posed by idiopathic cutaneous mastocytosis in infants, particularly in resource-limited settings where dermatological expertise and diagnostic tools may be inaccessible. The atypical presentation mimicking infantile psoriasis highlights the importance of having cutaneous mastocytosis as a differential diagnosis when evaluating children with suspected psoriasis and reliance on histopathological confirmation. Effective management, even in low-resource environments, can significantly improve patient outcomes with appropriate education and supportive care.

## Data Availability

All data supporting this case report are included in the article, with no additional data available due to patient confidentiality.
